# Supplementary material for: Are violence, harmful alcohol/substance use and poor mental health associated with increased genital inflammation?: A longitudinal cohort study with HIV-negative female sex workers in Nairobi, Kenya
Source: PLOS Glob Public Health. 2024 Aug 27;4(8):e0003592. doi: 10.1371/journal.pgph.0003592 (PMC11349110; doi:10.1371/journal.pgph.0003592)
Supplement: S1 Table — a: Exposure, outcome, and contextual variables. b: Assessment of pregnancy, STIs and HIV. (DOCX) [file pgph.0003592.s001.docx]

**Supporting Table 1a: Exposure, outcome, and contextual variables**

| Indicator | Variable construction |
| --- | --- |
| **OUTCOME** | |
| Genital inflammation | Binary: Inflammation; No inflammation  Coded as ‘Inflammation’ if at least 5 out of 9 of the following cytokines/chemokines are raised (above baseline median value for that cytokine):   - macrophage inflammatory protein [MIP]-1α - MIP-1β - interferon-γ inducible protein [IP]-10 - interleukin [IL]-8 - monocyte chemoattractant protein [MCP]-1 - IL1α - IL-1β - IL-6 - tumour necrosis factor [TNF] |
| **EXPOSURES** | |
| *Past 6 month physical violence* | Binary: Yes; No  Coded as ‘Yes’ if she reports that an intimate partner or anyone else has done at least one of the following things to her in the past 6 months:   - Pushed you, shaken you, or thrown something at you - Slapped or shoved you - Pushed you or shoved you or pulled your hair - Hit you with his fist or something else that could hurt you - Kicked you, dragged you or beat you up - Tried to choke or burn you on purpose - Threatened to use or actually used a gun, knife or other weapon |
| *Past 6 month sexual violence* | Binary: Yes; No  Coded as ‘Yes’ if she reports that an intimate partner or anyone else has done at least one of the following things to her in the past 6 months:   - Physically forced you to have sex with him even when you did not want to - Used threats of violence or rejection to force you to have sex with him when you did not want to - Forced you to do something sexual that you found degrading or humiliating   AND/OR  She answers ‘Yes’ to at least one of the following:   - Has there been a time in the past 7 days when you were physically forced to have sex with your intimate partner even when you did not want to? - Has there been a time in the past 7 days when you were physically forced to have sex with someone (other than your intimate partner) even when you did not want to? - Have you been gang-raped (in the past 6 months)? |
| Past 6 months physical/sexual violence | Categorical: None; Physical only; Sexual (with or without physical)  Composite of above two variables. |
| *Past 6 months moderate intensity emotional violence* | Binary: Yes; No  Coded as ‘Yes’ if she reports that an intimate partner or anyone else has ever, in the past 6 months:   - Threatened to hurt or harm you or someone close to you   AND/OR  that an intimate partner or anyone else has done at least two of the following things to her ever in the past 6 months:   - Said or done something to humiliate you in front of others - Insulted you repeatedly to make you feel bad about yourself - Done things to scare or intimidate you on purpose, e.g. by the way of looking at you, by yelling or smashing things |
| *Past 6 months high intensity emotional violence* | Binary: Yes; No  Coded as ‘Yes’ if she reports that an intimate partner or anyone else has ‘Many times’ in the past 6 months:   - Threatened to hurt or harm you or someone close to you   AND/OR  that an intimate partner or anyone else has done at least two of the following things to her ‘Many times’ in the past 6 months:   - Said or done something to humiliate you in front of others - Insulted you repeatedly to make you feel bad about yourself - Done things to scare or intimidate you on purpose, e.g. by the way of looking at you, by yelling or smashing things |
| Past 6 months emotional violence (ordered categorical) | Ordered categorical: None/low; Moderate intensity; High intensity  Composite of the above two variables (Coded as ‘None/low’ if *Moderate intensity emotional violence=No*; Coded as ‘Moderate intensity’ if *Moderate intensity emotional violence=Yes* and *High intensity emotional violence=No*; Coded as ‘High intensity’ if *High intensity emotional violence=Yes*) |
| Harmful alcohol use | Ordered categorical: Low risk; Moderate risk; High risk  Measured using WHO ASSIST (Alcohol, Smoking and Substance Involvement Screening Test) tool.  Coded as ‘Low risk’ if score <11; Coded as ‘Moderate risk’ if score 11-27; Coded as ‘High risk’ if score >27) |
| Other harmful substance use (excluding alcohol and tobacco) | Ordered categorical: Low risk; Moderate risk; High risk  Measured using WHO ASSIST (Alcohol, Smoking and Substance Involvement Screening Test) tool.  Coded as ‘Low risk’ if score <5; Coded as ‘Moderate risk’ if score 5-27; Coded as ‘High risk’ if score >27) |
| *Anxiety* | Ordered categorical: None; Mild; Moderate/Severe  Measured using Generalised Anxiety Disorder (GAD-7) tool.  Coded as ‘None’ if score 0-4; Coded as ‘Mild’ if score 5-9; Coded as ‘Moderate/Severe’ if score 10+ |
| *Depression* | Ordered categorical: None; Mild; Moderate/Severe  Measured using Patient Health Questionnaire (PHQ-9).  Coded as ‘None’ if score 0-4; Coded as ‘Mild’ if score 5-9; Coded as ‘Moderate/Severe’ if score 10+ |
| Anxiety and/or depression | Ordered categorical: None; Mild; Moderate/Severe  Composite of above two variables, with category corresponding to woman’s highest score for anxiety or depression. |
| PTSD | Binary: No PTSD; PTSD  Measured using Harvard Trauma Questionnaire (HTQ-17).  Coded as positive for PTSD if score of ≥2.0. |
| **POTENTIAL CONFOUNDERS ADJUSTED FOR IN ANALYSIS** | |
| Age | Ordered categorical: 0-24yrs; 25-34yrs; ≥35yrs |
| Current intimate partner | Binary: Yes; No  Coded as ‘Yes’ if currently has a non-paying intimate partner. |
| Education | Ordered categorical: Did not complete primary; Completed primary (but not secondary); Completed secondary or higher |
| Female genital mutilation | Binary: Yes; No  Coded as ‘Yes’ if answers yes to:  ‘Have you ever had a surgical procedure used for modifying the vagina, or restoration of the hymen; including female genital circumcision, incision with insertion of substance into the lesion (scarification process, tattoos of the vulva or labia)?’ |
| Intravaginal washing practices | Categorical: None; Water only; Other substances  Coded as ‘None’ if answer no to “In the past 30 days, have you cleaned inside the vagina (beyond the outside opening)?”;  Coded as ‘Water only’ if answer yes to having cleaned inside the vagina in past 30 days, and report using water only in response to multiple choice question “What did you clean with?”  Coded as ‘Other substances’ if answer yes to having cleaned inside the vagina in past 30 days, and report using soap, shower gel, household cleaners, antiseptic solutions, vinegar, lemon juice or other substances. |
| Tobacco use (past 3 months) | Binary: Any; None  Coded as ‘Any’ if choose any response frequency except ‘Never’ or ‘No answer’ in response to “In the past 3 months, how often have you used tobacco?” |

**Supporting Table 1b: Assessment of pregnancy, STIs and HIV**

| **Method of assessment** | **Health condition(s) assessed** |
| --- | --- |
| Urine sample | - Pregnancy - Chlamydia trachomatis (CT) (GeneXpert Assay) - Neisseria gonorrhoeae (NG) infection (GeneXpert Assay) |
| Blood test | - Treponema pallidum (syphilis), defined as a positive RPR of any titre, using the rapid plasma regain assay - HIV (Rapid test, positive tests confirmed using HIV DNA GeneXpert) |
| Self-collected vaginal swabs | - Bacterial vaginosis (BV; Gram’s stain and Nugent scoring) - Trichomonas vaginalis (TV; OSOM Trichomonas Rapid Test; SEKISUI Diagnostics, LLC) |
