# Supplementary material for: Are violence, harmful alcohol/substance use and poor mental health associated with increased genital inflammation?: A longitudinal cohort study with HIV-negative female sex workers in Nairobi, Kenya
Source: PLOS Glob Public Health. 2024 Aug 27;4(8):e0003592. doi: 10.1371/journal.pgph.0003592 (PMC11349110; doi:10.1371/journal.pgph.0003592)
Supplement: S2 Table — a. Results of sensitivity analysis—Odds ratios of association between baseline exposure variables and baseline genital inflammation outcome as defined by Masson et al. (2015) using upper quartile as the threshold to define raised levels of each cytokine. b. Results of sensitivity analysis—Odds ratios of association between trajectory of exposure variables and genital inflammation at follow-up, as defined by Masson et al. (2015) using upper quartile as the threshold to define raised levels of each cytokine. (DOCX) [file pgph.0003592.s002.docx]

Supporting Table 2a. Results of sensitivity analysis - Odds ratios of association between baseline exposure variables and baseline genital inflammation outcome as defined by Masson et al. (2015) using upper quartile as the threshold to define raised levels of each cytokine.

| Baseline exposures |  | n (weighted %)  N=727 | Weighted % with genital inflammation at baseline | OR (95% CI)  [Adjusted Wald test p-value] | aOR* (95%CI)  [Adjusted Wald test p-value] |
| --- | --- | --- | --- | --- | --- |
| **Violence exposures (past 6 months by any perpetrator)** |  |  |  |  |  |
| Experience of physical and sexual violence | None | 241 (32%) | 20% | - | - |
|  | Physical only | 108 (14%) | 18% | 0.87 (0.48 – 1.55) | 0.81 (0.43 – 1.51) |
|  | Sexual (with or without physical) | 378 (53%) | 16% | 0.76 (0.50 – 1.15) | 0.75 (0.49 – 1.15) |
|  |  |  | p=0.429 | [p=0.435] | [p=0.410] |
| Emotional violence | None | 287 (39%) | 20% | - | - |
|  | Moderate | 310 (44%) | 18% | 0.86 (0.58 – 1.30) | 0.85 (0.56 – 1.30) |
|  | Severe | 130 (18%) | 12% | 0.53 (0.29 – 0.98) | 0.48 (0.26 – 0.89) |
|  |  |  | p=0.122 | [p=0.128] | [p=0.067] |
| **Alcohol and substance abuse (past 3 months)** |  |  |  |  |  |
| Alcohol ASSIST risk level | Low (0-10) | 474 (66%) | 20% | - | - |
|  | Moderate (11-26) | 169 (23%) | 17% | 0.84 (0.53 – 1.32) | 0.86 (0.53 – 1.39) |
|  | High (27+) | 80 (11%) | 9% | 0.42 (0.19 – 0.93) | 0.44 (0.19 – 1.03) |
|  |  |  | p=0.079 | [p=0.093] | [p=0.156] |
| Other substance use ASSIST risk level (not alcohol or tobacco) | Low | 531 (74%) | 20% | - | - |
|  | Moderate/High | 196 (26%) | 12% | 0.53 (0.33 – 0.86) | 0.53 (0.31 – 0.91) |
|  |  |  | p=0.009 | [p=0.010] | [p=0.022] |
| **Mental health exposures** |  |  |  |  |  |
| Anxiety and/or depression (past 2 weeks) | Low | 341 (46%) | 17% | - | - |
|  | Mild | 209 (29%) | 21% | 1.37 (0.89 – 2.12) | 1.43 (0.90 – 2.27) |
|  | Moderate/severe | 177 (25%) | 16% | 0.95 (0.58 – 1.55) | 1.07 (0.63 – 1.80) |
|  |  |  | p=0.261 | [p=0.263] | [p=0.289] |
| PTSD (past month) | No | 621 (86%) | 18% | - | - |
|  | Yes | 99 (14%) | 15% | 0.80 (0.45 – 1.43) | 0.91 (0.50 – 1.66) |
|  |  |  | p=0.458 | [p=0.459] | [p=0.760] |

*Adjusted for age, education, current intimate partner, FGM, intravaginal washing practices, smoking and SWOP clinic

Supporting Table 2b. Results of sensitivity analysis - Odds ratios of association between trajectory of exposure variables and genital inflammation at follow-up, as defined by Masson et al. (2015) using upper quartile as the threshold to define raised levels of each cytokine

|  | n (weighted %)  N=538 | Weighted % with genital inflammation at follow-up | OR (95% CI)  [Adjusted Wald test p-value] | aOR* (95%CI)  [Adjusted Wald test p-value] |
| --- | --- | --- | --- | --- |
| **Sexual violence (with or without physical violence)** |  |  |  |  |
| Never | 232 (42%) | 19% | - | - |
| Baseline only | 200 (38%) | 16% | 0.82 (0.50 – 1.35) | 0.79 (0.47 – 1.33) |
| Follow-up (with or without baseline) | 106 (20%) | 12% | 0.56 (0.29 – 1.09) | 0.60 (0.30 – 1.19) |
|  |  | p=0.225 | [p=0.226] | [p=0.320] |
| **Moderate/severe emotional violence** |  |  |  |  |
| Never | 175 (32%) | 18% | - | - |
| Baseline only | 203 (38%) | 17% | 0.92 (0.54 – 1.58) | 0.92 (0.52 – 1.60) |
| Follow-up (with or without baseline) | 160 (30%) | 16% | 0.87 (0.49 – 1.53) | 0.85 (0.47 – 1.55) |
|  |  | p=0.884 | [p=0.884] | [p=0.873] |
| **Moderate/high risk alcohol use** |  |  |  |  |
| Never | 321 (60%) | 17% | - | - |
| Baseline only | 135 (25%) | 17% | 1.06 (0.62 – 1.80) | 1.10 (0.63 – 1.92) |
| Follow-up (with or without baseline) | 82 (15%) | 16% | 0.97 (0.50 – 1.88) | 1.11 (0.52 – 2.36) |
|  |  | p=0.966 | [p=0.966] | [p=0.929] |
| **Moderate/high risk substance use** |  |  |  |  |
| Never | 339 (65%) | 19% | - | - |
| Baseline only | 61 (11%) | 16% | 0.81 (0.39 – 1.66) | 0.82 (0.38 – 1.76) |
| Follow-up (with or without baseline) | 138 (25%) | 12% | 0.57 (0.32 – 1.02) | 0.55 (0.26 – 1.13) |
|  |  | p=0.145 | [p=0.159] | [p=0.266] |
| **Moderate/severe anxiety and/or depression** |  |  |  |  |
| Never | 381 (69%) | 18% | - | - |
| Baseline only | 105 (20%) | 17% | 0.92 (0.52 – 1.65) | 0.80 (0.43 – 1.47) |
| Follow-up (with or without baseline) | 52 (11%) | 11% | 0.57 (0.24 – 1.38) | 0.57 (0.23 – 1.42) |
|  |  | p=0.452 | [p=0.462] | [p=0.424] |
| **PTSD** |  |  |  |  |
| Never | 464 (86%) | 17% | - | - |
| Baseline only | 64 (12%) | 19% | 1.14 (0.59 – 2.21) | 1.29 (0.67 – 2.49) |
| Follow-up (with or without baseline) | 10 (2%) | 11% | 0.63 (0.08 – 4.68) | 0.60 (0.09 – 4.20) |
|  |  | p=0.823 | [p=0.827] | [p=0.640] |

*Adjusted for age, education, current intimate partner, FGM, intravaginal washing practices, smoking and SWOP clinic
