# Supplementary material for: Are violence, harmful alcohol/substance use and poor mental health associated with increased genital inflammation?: A longitudinal cohort study with HIV-negative female sex workers in Nairobi, Kenya
Source: PLOS Glob Public Health. 2024 Aug 27;4(8):e0003592. doi: 10.1371/journal.pgph.0003592 (PMC11349110; doi:10.1371/journal.pgph.0003592)
Supplement: Attachment — Submitted filename: Responses to authors-PLOS Global Public Health 2024.docx [file pgph.0003592.s003.docx]

| Number | Reference /Line | Reviewer comments |
| --- | --- | --- |
| 1. | Title | The title of this paper, “*Are violence, harmful alcohol/substance use and poor mental health associated with increased genital inflammation?: A longitudinal cohort study with HIV-negative Female Sex Workers in Nairobi, Kenya”,* is informative. It only needs to be narrowed and specify the target.  The author may say ”*Harmful substance use and mental health related violence to increase genital inflammation: Case of Female sex workers in Kenya*” |
| 2. | **Abstract**  **Introduction: Line 25-29**  **Methods:**  **30-37**  **Results: 38-44** | The introduction combines one part of the methodology “*We examined whether past 6-month experience of these exposures was associated with increased genital inflammation, thereby providing a biological link between these exposures and HIV acquisition risk, among a cohort of female sex workers (FSWs) in Nairobi, Kenya*”  The introduction can only provide what is known and the existing gaps.  The design is clear enough and types of measures. Setting, sample size and sampling strategy is not clear. Also, the tool for analysis or software is not available which may be of interest to add them and help readers to understand for the first read (abstract).  Good presentation of the results. You may consider not adding numbers and percentage in this section as it just an abstract  **General comments on the abstract**: It is well structured and informative. Consider to reaffirm the comments given and see how to not include abbreviations in the abstract |
| 3. | **Introduction:**  **20-27** | Justifies the purpose of the research whereby showing the exact gaps found.  You can also consider expanding and explain the meaning of substance use as you described the alcohol. For example, in substance use, if you think tobacco is part of, now go ahead and explain what tobacco does on the body of the same population. And so on…  This Maisha fiti study can be explained in the methodology part rather than in the introduction. In addition, you can conclude your background with a statement of the study purpose instead. |
| 4 | **Methods**  **29-36: The study setting**  **37-39: The design**  **39-207:**  **209-234: Laboratory methods:**  **220:** | The study design usually come first and you may add the information from the other Maisha Fiti you used.  For this study setting, It is clear that the city of Nairobi is sensitive. You only need to convince readers your choice in a comparison of 2 to 3 counties of Kenya and the prevalence of these. Otherwise, the prevalence may be counted as national data.  The study design was not well explained and why you choose that one. The presented one was the ideas from the team and should have a scientific reason to be selected. Example, longitudinal behavioral-biological design…  There is a huge information with no specification of the following:   - **Population**: Is there and well defined though it is combined with other steps - **Sample size and calculation**: Well explained with some mixed-up of information - **Sampling strategy**: Also it was explained with too much information - **Recruitment plan**: Explained in a vague way - **Data collection**: Explained with recruitment   There is a need to specify the provided information under these sub-titles to make a coherent message  This becomes like a new study (RCT) or a new design (Experimental design). The information may be considered with the explanation of the biological factors (aspect) to show readers that it falls into your planed design  The same with sample collection at multiple times. This should clearly be explained and show that there was no harm to the subjects (population or women)  This brings in analysis method and may be considered as subtopic. Also explain why you choose this type of analysis and the used software (SPSS, STATA,….) and why |
| **5** | **235-246** | Conceptual framework:  The developed framework does not explain the basis of it (what framework informed the development of it) in the description. It is crucial to show and explain the steps you passed through while developing this as well |
| 6 | **248-275: Analysis**  **277-281: Ethics statement:** | There was a repetitive information on the analysis and it would be better if you explain it in single area like here. It is clear now that you explained the used software (STATA 16.1). Explain whether you did secondary data analysis or primary or both as you include Maisha Fiti throughout.  This statement is important. It can be added directly under recruitment plan before data collection |
| 7 | **283-301: Results** | There is inconsistent in number of study population. On the line 142 under study design and sampling, the eligibility covered 10,292 women. Now, in the results section, they become 1200. The difference should be explained and be cleared.  You may have considered response rate per data. Kindly explain it  The tables (1,2 and 3) are well presented and the information under them is well structured. You only need to present, in the statement, a few information as the table speaks itself. |
| **8** | **385-449: Conclusion** | Conclusion combines a huge information which may be separated such as strength of the study and the weakness of area that needs more exploration. Kindly review and summarize your conclusion with the main points |

**General comments**

This is a very great job done by the team. The paper reads well and provide enough information for readers.

**Only some points to improve are given in the table in addition to**:

- The results were not discussed with the supporting evidence. That means, no discussion part is available. It can be easily added as the team can.
- The strengths of the project as well as rationale of it
- You may also add each author’s contribution to this paper

**Great job!!!!!!!!!!!!!!!!!!**
