# Supplementary material for: Are violence, harmful alcohol/substance use and poor mental health associated with increased genital inflammation?: A longitudinal cohort study with HIV-negative female sex workers in Nairobi, Kenya
Source: PLOS Glob Public Health. 2024 Aug 27;4(8):e0003592. doi: 10.1371/journal.pgph.0003592 (PMC11349110; doi:10.1371/journal.pgph.0003592)
Supplement: Attachment — Submitted filename: Response to Reviewers.docx [file pgph.0003592.s004.docx]

June 25, 2024

Dear Dr. Bagade,

On the behalf of my coauthors, I would like to sincerely thank the editorial board and peer reviewers for their thoughtful review of our manuscript titled “Are violence, harmful alcohol/substance use and poor mental health associated with increased genital inflammation?: A longitudinal cohort study with HIV-negative Female Sex Workers in Nairobi, Kenya” (manuscript number: PGPH-D-24-00503).

Most importantly, both reviewers suggested that more details on the sample size calculation used for this study were needed, which we have now included in the Methods section (lines 165 –176) . In addition to minor grammatical and formatting changes throughout the manuscript, we have also added more important background information to the Introduction section (lines 109 –115) and inserted a new Methods subsection that outlines our recruitment plan (lines 175 –186).

We believe that these revisions, outlined in detail in the point-by-point response below, have substantially improved the manuscript. We hope that the editorial board and peer reviewers agree, and that the manuscript is now acceptable for publication in PLoS Global Public Health.

Sincerely,

James Pollock

-----------------------------------------------------------

**Reviewer #1:**

This is a very innovative study for public health implication. The analysis was thoroughly done, although the overall justification for the study was not adequately discussed.

First, the topic seems inappropriate, especially just mentioning “genital inflammation” this is non specific of a particular or specific diagnosis or infection. It should be stated what exactly the outcome of study is, for example, genital inflammation associated with HIV seroconversion or infection.

***The outcome of the study is a composite inflammation score that was previously validated with HIV acquisition as an outcome. We have now added more detail regarding this genital inflammation composite score and the rationale behind it (lines 303 - 313).***

Secondly, the conclusion in the abstract states that ...this HIV acquisition risk is more fully explained by behavioural rather than biological pathways. The researcher should be careful of the use of the word fully. This seems inappropriate, no study or findings of a research fully explains the outcome.

***The reviewer makes an excellent point. We have revised the conclusions section of the abstract to better reflect the findings of this study, as follows (lines 45 – 50):***

***We find no association between past 6 month experience of violence, harmful alcohol/substance use, poor mental health, and immune parameters previously associated with HIV risk. This suggests that the well-described epidemiological associations do not appear to be mediated by genital immune changes, or that any changes are relatively short-lived. High prevalences of these exposures in this setting suggest an urgent need for sex-worker specific violence, alcohol/substance use and mental health interventions.***

Third; line 105 to 110, the explanation on the relationship between alcohol, condomless sex, genital inflammation, and HIV susceptibility is not clear. Is the researcher trying to attribute drinking and condomless sex to genital inflammation? yes alcohol could impair sexual-decision making to engage in condomless sex, and it can decrease immunity but what is the link between these and genital inflammation?

***Apologies for any lack of clarity. We do not wish to attribute drinking and condomless sex to genital inflammation. Our group has previously found that any receptive penile-vaginal sex causes a transient increase in genital inflammation. Since harmful alcohol use increases the likelihood of condomless sex, this may be a pathway for harmful alcohol use to increase genital inflammation, and thus HIV susceptibility if the male partner lives with HIV. This explanation has been revised in the Introduction section (lines 108 – 109).***

Line 113, remove “...with bi-directional associations” the relationship between those variables are all not bi-directional. Also your example is inappropriate, why use the words “women who are drunk”...you should rather use articles from past studies to butress your point with correct citation.

***We have adjusted the language used in this section and cited an additional systematic review that describes the bi-directional relationship between substance use and mental health disorders (line 118). We hope that the citation of three systematic reviews that describe the directionality of associations between these three variables better establishes our statement on line 113.***

Line 142; not appropriate “10,292/29,000” does this means 10,292 divides by 29,000? Be clearer.

***We mean to say, “10,292 of 29,000 participants”. We have now reworded this sentence for clarity (line 142).***

Line 146; why 1000 FSWs? what informs the sample size determination?

***Details on the sample size calculation have now been added, as follows (lines 163 – 168):***

***To detect a 10% difference in the proportion of women who have genital inflammation (25% vs. 15%) at 90% power, 750 HIV-negative women were enrolled, assuming 2:1 exposure of recent sexual or physical violence. As HIV prevalence among FSWs attending SWOP clinics in Nairobi is approximately 25%, the target sample size was 1000 FSWs. Oversampling was used to allow for non-eligibility and non-response – thus 1200 women were randomly selected for participation.***

The justification for this study was not clearly explained. Alcohol use is associated with Sexual violence from previous studies, it is also established that sexual violence increases vulnerability to HIV however, the number of HIV incidence from sexual violence is low and also linking all this variables together was not clearly explained in this study.

***We hope the re-wording of the study rationale clarifies the biological premise of this paper.***

The researcher should have a discussion section to explicitly discuss the findings and relate to previous studies done relating this variables for clarity.

***Our original manuscript included all discussion of findings in the “Conclusions” section. We have now re-named the section to “Discussion” and separated the discussion, strengths, limitation, and conclusions into individual paragraphs.***

**Reviewer #2:**

1. Title

The title of this paper, “Are violence, harmful alcohol/substance use and poor mental health associated with increased genital inflammation?: A longitudinal cohort study with HIV-negative Female Sex Workers in Nairobi, Kenya”, is informative. It only needs to be narrowed and specify the target.

The author may say ”Harmful substance use and mental health related violence to increase genital inflammation: Case of Female sex workers in Kenya”

***While we appreciate that a more concise title has advantages, the precise title suggested seems a little too restrictive. We not only discuss mental health related violence, but physical and sexual violence as well, which can be quite distinct. We have discussed this within our team and would like our original title to remain unchanged.***

2. Abstract

Introduction: Line 25-29

The introduction combines one part of the methodology “We examined whether past 6-month experience of these exposures was associated with increased genital inflammation, thereby providing a biological link between these exposures and HIV acquisition risk, among a cohort of female sex workers (FSWs) in Nairobi, Kenya”

The introduction can only provide what is known and the existing gaps.

***This statement has been moved to the Methods section of the abstract (lines 29 – 30).***

Methods: 30-37

The design is clear enough and types of measures. Setting, sample size and sampling strategy is not clear. Also, the tool for analysis or software is not available which may be of interest to add them and help readers to understand for the first read (abstract).

***After the re-wording of the Introduction and Methods sections, given the reviewer’s previous comment, we hope that these details are now more clear.***

Results: 38-44

Good presentation of the results. You may consider not adding numbers and percentage in this section as it just an abstract

***Thank your for the suggestion, however we would prefer to include those details for completeness.***

General comments on the abstract:

It is well structured and informative. Consider to reaffirm the comments given and see how to not include abbreviations in the abstract

***Thank you for your kind words. We have now written out the abbreviations “FSWs” and “PTSD” in full.***

3. Introduction:

Justifies the purpose of the research whereby showing the exact gaps found. You can also consider expanding and explain the meaning of substance use as you described the alcohol. For example, in substance use, if you think tobacco is part of, now go ahead and explain what tobacco does on the body of the same population. And so on…

***The reviewer makes an important point. Several studies have explored inflammatory biomarkers in people that use tobacco, cocaine, and cannabis, finding that substance use can cause immune activation or immunosuppression, depending on the substance, immune parameter, and degree of substance use. We have now added a discussion of the immunological effects of tobacco and other substance use (lines 111 – 114).***

20-27

This Maisha fiti study can be explained in the methodology part rather than in the introduction. In addition, you can conclude your background with a statement of the study purpose instead.

***We agree that the last paragraph of the Introduction was rich in methodology; this has been moved to the Methods section (lines 133 – 135).***

4. Methods

29-36: The study setting

The study design usually come first and you may add the information from the other Maisha Fiti you used.

For this study setting, It is clear that the city of Nairobi is sensitive. You only need to convince readers your choice in a comparison of 2 to 3 counties of Kenya and the prevalence of these. Otherwise, the prevalence may be counted as national data.

***The details of the Maisha Fiti study design have been moved to the first part of the Methods section. The reviewer asks for a justification of Nairobi as our study setting. We’ve now added some statistics of three Kenyan counties with high HIV incidence, including Nairobi county, and the prevalence of violence and mental health issues among female sex workers in Nairobi (lines 139 – 144).***

37-39: The design

The study design was not well explained and why you choose that one. The presented one was the ideas from the team and should have a scientific reason to be selected. Example, longitudinal behavioral-biological design…

***The reviewer requested a better justification of our study design. The longitudinal design allows us to assess any causal relationship between violence, mental health, and alcohol/substance use and genital inflammation. We have now addressed this in the Methods section (lines 133 – 155).***

39-207:

There is a huge information with no specification of the following:

- Population: Is there and well defined though it is combined with other steps

- Sample size and calculation: Well explained with some mixed-up of information

- Sampling strategy: Also it was explained with too much information

- Recruitment plan: Explained in a vague way

- Data collection: Explained with recruitment

There is a need to specify the provided information under these sub-titles to make a coherent message

***We have added details on the participant recruitment plan and revised the Methods section to better align with the proposed structure.***

209-234: Laboratory methods:

This becomes like a new study (RCT) or a new design (Experimental design). The information may be considered with the explanation of the biological factors (aspect) to show readers that it falls into your planed design The same with sample collection at multiple times. This should clearly be explained and show that there was no harm to the subjects (population or women)

***We now provide more details of the planned design and sampling timeframe, as well as procedures intended to reduce harm among participants (lines 175 – 183). Several considerations were made to mitigate the risk of harm to participants. The biggest risk to participants in this study was the emotional trauma that is sometimes associated with recounting experiences of violence and abuse. To address this, all participants were offered referral to on-site psychological counsellors, regardless of what they had disclosed in their interviews.***

220:

This brings in analysis method and may be considered as subtopic. Also explain why you choose this type of analysis and the used software (SPSS, STATA,….) and why

***This description of the analysis has now been moved under the “Statistical analysis” subheading (lines 262 – 304).***

5. 235-246

Conceptual framework: The developed framework does not explain the basis of it (what framework informed the development of it) in the description. It is crucial to show and explain the steps you passed through while developing this as well

***The conceptual framework used a structural determinants and eco-social perspective. These theories describe how structural and social determinants or ‘exposures beyond an individual’s control’ (e.g. violence experience) may become biologically integrated (e.g. genital inflammation) through direct (e.g. rape) and/or indirect (e.g. cortisol induced dysregulation of the HPA) pathways. The framework was informed by global reviews of the current violence against women, harmful alcohol/substance, mental health and HIV literatures, which identified three potential pathways through which the exposures (violence, harmful alcohol/substance use, mental health) may influence the outcome (genital inflammation). The literature reviews also identified potential confounders and effect modifiers. We have now included this detail in the Methods section (lines 235 – 267).***

6. 248-275: Analysis

There was a repetitive information on the analysis and it would be better if you explain it in single area like here. It is clear now that you explained the used software (STATA 16.1). Explain whether you did secondary data analysis or primary or both as you include Maisha Fiti throughout.

***We have now consolidated the analysis information into this section. All analyses presented in this paper are primary.***

277-281: Ethics statement:

This statement is important. It can be added directly under recruitment plan before data collection

***The suggested revision has been made.***

7. 283-301: Results

There is inconsistent in number of study population. On the line 142 under study design and sampling, the eligibility covered 10,292 women. Now, in the results section, they become 1200. The difference should be explained and be cleared.

You may have considered response rate per data. Kindly explain it

The tables (1,2 and 3) are well presented and the information under them is well structured. You only need to present, in the statement, a few information as the table speaks itself.

***We have now added additional details to the sample size subsection in order to clarify the reviewer’s sampling questions.***

8. 385-449: Conclusion

Conclusion combines a huge information which may be separated such as strength of the study and the weakness of area that needs more exploration. Kindly review and summarize your conclusion with the main points

***The discussion of study strengths and limitations has now been separated into two paragraphs (lines 446 – 465).***

General comments

This is a very great job done by the team. The paper reads well and provide enough information for readers.

Only some points to improve are given in the table in addition to:

- The results were not discussed with the supporting evidence. That means, no discussion part is available. It can be easily added as the team can.

- The strengths of the project as well as rationale of it

- You may also add each author’s contribution to this paper

Great job!!!!!!!!!!!!!!!!!

***Thank you for your kind comments. Author contributions were uploaded to the Editorial Manager system for PLOS Global Public Health.***
